# Down-regulation of EPB41L4A-AS1 mediated the brain aging and neurodegenerative diseases via damaging synthesis of NAD^+^ and ATP

**DOI:** 10.1186/s13578-021-00705-2

**Published:** 2021-11-10

**Authors:** Tingpeng Yang, Yanzhi Wang, Weijie Liao, Shikuan Zhang, Songmao Wang, Naihan Xu, Weidong Xie, Cheng Luo, Yangyang Wang, Ziqiang Wang, Yaou Zhang

**Affiliations:** 1 Department of Chemical Engineering, Tsinghua University, Beijing, 100084 People’s Republic of China; 2 State Key Laboratory of Chemical Oncogenomics, Tsinghua Shenzhen International Graduate School, Shenzhen, 518055 China; 3 School of Life Sciences, Tsinghua University, Beijing, 100084 China; 4 Key Lab in Healthy Science and Technology of Shenzhen, Tsinghua Shenzhen International Graduate School, Shenzhen, 518055 China; 5 Open FIESTA Center, Tsinghua Shenzhen International Graduate School, Tsinghua University, Shenzhen, 518055 China; 6 Biomedical Sciences College & Shandong Medicinal Biotechnology Centre, Shandong First Medical University & Shandong Academy of Medical Sciences, Jinan, 250062 China; 7 Department of Biomedical Engineering, Tsinghua University, Beijing, 100084 China

**Keywords:** Aging, Neurodegenerative diseases, EPB41L4A- AS1, NAD^+^, ATP, H3K27Ac

## Abstract

**Background:**

Aging and neurodegenerative diseases are typical metabolic-related processes. As a metabolism-related long non-coding RNA, EPB41L4A-AS has been reported to be potentially involved in the development of brain aging and neurodegenerative diseases. In this study, we sought to reveal the mechanisms of EPB41L4A-AS in aging and neurodegenerative diseases.

**Methods:**

Human hippocampal gene expression profiles downloaded from the Genotype-Tissue Expression database were analyzed to obtain age-stratified differentially expressed genes; a weighted correlation network analysis algorithm was then used to construct a gene co-expression network of these differentially expressed genes to obtain gene clustering modules. Gene Ontology, Kyoto Encyclopedia of Genes and Genomes, protein–protein interaction network, and correlation analysis were used to reveal the role of EPB41L4A-AS1. The mechanism was verified using Gene Expression Omnibus dataset GSE5281 and biological experiments (construction of cell lines, Real-time quantitative PCR, Western blot, measurement of ATP and NAD^+^ levels, nicotinamide riboside treatment, Chromatin Immunoprecipitation) in neurons and glial-derived cells.

**Results:**

EPB41L4A-AS1 was downregulated in aging and Alzheimer's disease. EPB41L4A-AS1 related genes were found to be enriched in the electron transport chain and NAD^+^ synthesis pathway. Furthermore, these genes were highly associated with neurodegenerative diseases and positively correlated with EPB41L4A-AS1. In addition, biological experiments proved that the downregulation of EPB41L4A-AS1 could reduce the expression of these genes via histone H3 lysine 27 acetylation, resulting in decreased NAD^+^ and ATP levels, while EPB41L4A-AS1 overexpression and nicotinamide riboside treatment could restore the NAD^+^ and ATP levels.

**Conclusions:**

Downregulation of EPB41L4A-AS1 not only disturbs NAD^+^ biosynthesis but also affects ATP synthesis. As a result, the high demand for NAD^+^ and ATP in the brain cannot be met, promoting the development of brain aging and neurodegenerative diseases. However, overexpression of EPB41L4A-AS1 and nicotinamide riboside, a substrate of NAD^+^ synthesis, can reduce EPB41L4A-AS1 downregulation-mediated decrease of NAD^+^ and ATP synthesis. Our results provide new perspectives on the mechanisms underlying brain aging and neurodegenerative diseases.

**Supplementary Information:**

The online version contains supplementary material available at 10.1186/s13578-021-00705-2.

## Introduction

Long non-coding RNAs (lncRNAs) are a class of non-coding RNAs usually longer than 200 nucleotides in length [1]. For a long time, lncRNAs have been regarded as useless cell components; however, in recent years, an increasing number of studies have shown that lncRNAs played important regulatory roles in cells. It has been reported that lncRNAs are involved in many processes, such as the MEK/ERK signaling pathway [2], ferroptosis [3], metastasis of clear cell renal cell carcinoma [4], and the occurrence of cancers [5, 6]. Furthermore, lncRNAs have been documented to play an important regulatory role in cell metabolism, including glycolysis [7] and Aβ clearance [8].

EPB41L4A-AS1 was first reported as a protein-coding gene named TIGA1 related to cell proliferation [9]; later studies demonstrated that EPB41L4A-AS1 mainly functioned as a lncRNA associated with cell metabolism and immune response [7, 10, 11], and its downregulation in cancer cells induced glutamine dependency and promoted glycolysis [7]. Interestingly, it has been shown that downregulation of EPB41L4A-AS1 in placental trophoblast cells could inhibit the Warburg effect essential for fast growth of placental trophoblast cells leading to recurrent miscarriages [10]. Furthermore, downregulation of EPB41L4A-AS1 could reportedly activate the MYD88-Dependent NF-κB pathway in diabetes-related inflammation [11].

Intriguingly, the decline of brain energy metabolism is an important cause of the age-associated functional decline and brain disease and can subtly appear even before the diagnosis of neurodegenerative diseases [12]. Mitochondrial dysfunction is the main cause of the decline of brain energy metabolism in the aging brain and neurodegenerative diseases. In this regard, it is widely acknowledged that NAD^+^ depletion contributes to mitochondrial dysfunction, reducing ATP production, the main currency of brain energy metabolism, leading to brain dysfunction [13–15]. NAD^+^ is not only a key coenzyme in energy metabolism but also a cofactor or substrate of hundreds of enzymes, playing multiple roles in DNA damage repair, gene expression, and stress response [13, 16, 17]. Therefore, the decline of NAD^+^ during aging influences many key cellular functions and induces many aging-associated diseases, including neurodegenerative diseases [16, 18]. However, the mechanisms underlying NAD^+^ depletion in the aging brain and neurodegenerative diseases and the role of lncRNAs remain unclear.

In the present study, we found that EPB41L4A-AS1 expression gradually decreased with aging. Given that EPB41L4A-AS1 plays an important role in metabolism, we hypothesized that EPB41L4A-AS1 downregulation exerted an important effect on brain metabolism and may be related to the occurrence of aging and neurodegenerative diseases. Our biological experiments showed that dysregulated expression of EPB41L4A-AS1 induced metabolic damage, presenting as a decrease of NAD^+^ and ATP synthesis. The downregulation of EPB41L4A-AS1 mediated the development of brain aging and neurodegenerative diseases, while EPB41L4A-AS1 overexpression could reduce the clinical symptoms. Importantly, our results provide new insights into the mechanisms of brain aging and neurodegenerative diseases.

## Materials and methods

### Data sets and bioinformatics analysis

Two datasets were used during the bioinformatics analysis. The GTEx (Genotype-Tissue Expression) database includes the gene expression data from 54 normal tissues. The gene expression data of 13 brain regions and corresponding information including age, gender, tissue location were extracted. GEO dataset GSE5281 was used to validate our proposed theory in Alzheimer's disease.

Microarray analysis was used to identify differentially expressed genes (DEGs), which often have important biological functions. R packages edgeR [19] and limma were used in this process. The required input data included the gene expression matrix and sample grouping matrix. In this study, samples were stratified according to their ages to obtain age-stratified DEGs.

WGCNA (weighted correlation network analysis) was used to perform cluster analysis and classify genes with similar expression patterns into the same module. Besides, the R package WGCNA was used to select the most relevant modules for the target trait (age) by analyzing sample traits of modules [20].

GO (Gene Ontology) [21] and KEGG (Kyoto Encyclopedia of Genes and Genomes) [22] enrichment analyses were performed to identify the biological functions and pathways of the identified target genes. The target genes could be involved in many functions or pathways; however, the significance of each function or pathway was assessed based on the gene ratios and P-values.

Correlation analysis was conducted to assess the strength of the correlation between EPB41L4A-AS1 and the target genes using the Pearson correlation analysis, and differences in the expression of EPB41L4A-AS1 in different age groups and between normal and AD groups were assessed using the Mann–Whitney U.

STRING (https://www.string-db.org/) was used to generate a protein–protein interaction (PPI) network to exhibit the interactions between proteins, including interactions from text mining, experiments, databases, co-expression, neighborhood, gene fusion, and co-occurrence [23].

### Cell lines and transfection

SH-SY5Y (ATCC, CL-0208) and U251 (ATCC, CL-0237) cells were cultured in DMEM/F 12 medium (Thermo Fisher Scientific) or DMEM medium (Thermo Fisher Scientific) with 10% FBS (Thermo Fisher Scientific) in a humidified incubator with 5% CO2 at 37 ℃. SiRNAs for EPB41L4A-AS1, negative control siRNA vectors, lentivirus carrying EPB41L4A-AS1 RNAi, negative control shRNA vectors, EPB41L4A-AS1 overexpression plasmids, and empty plasmids used as negative control were purchased from GenePharma (Suzhou, China). For transient transfection, siRNAs for EPB41L4A-AS1 and EPB41L4A-AS1 overexpression plasmids were transfected using Lipofectamine™ 2000 Transfection Reagent (Thermo Fisher Scientific), according to the manufacturer's protocol. U251 and SH-SY5Y cells were transfected with shEPB41L4A-AS1 plasmid to obtain stable knockdown cell lines, followed by 1 µg/mL final concentration of puromycin (Thermo Fisher Scientific) selection.

### Real-time quantitative PCR

RNAiso Plus (Takara) was used to extract total RNA. Reverse transcription of the total RNA was performed by One-Step RT-PCR SuperMix (TransGen Biotech). PerfectStartTM Green qPCR SuperMix (TransGen Biotech) was used to perform Real-time qRT-PCR. All mRNA levels were measured from three independent experiments and normalized to ACTB. Primers used for Real-time quantitative PCR are shown in Additional file 4: Table S1.

### Western blot

Proteins were extracted from cultured cells by cell extract buffer (50 mM Tris–HCl, pH 8.0, 4 M urea, and 1% Triton X-100) with protease inhibitor mixture (Roche Diagnostics, Cat.No:04693132001). Cell lysates were resolved by SDS-PAGE and then analyzed by western blotting. The following antibodies were used: ACTB (Proteintech, #20536–1-AP, 1:5000), NMNAT2 (Abnova, #H00023057-M04, 1:1000), NDUFS4 (Novus, #NBP1-31465, 1:1000), NDUFS6 (Novus, #NBP1-49831, 1:1000).

### Measurement of ATP and NAD^+^ levels

ATP and NADH levels were measured using Enhanced ATP Assay Kit (Beyotime Biotechnology) and Amplite™ Colorimetric NAD/NADH Ratio Assay Kit (AAT Bioquset), respectively, according to the manufacturer's protocol.

### NR (nicotinamide riboside) treatment for cells

NR was purchased from MCE (#HY-123033). Cells were cultured in a medium containing 100 mM NR after adhesion; follow-up experiments were carried out 24 h later. Cells cultured in the medium without NR were used as controls.

### ChIP (Chromatin Immunoprecipitation) assay

The ChIP assay was conducted as described previously in [8]. In brief, the cells were fixed with 1% formaldehyde and sonicated to shear the DNA. After centrifugation, the supernatants were incubated with H3K27Ac (Abcam, ab4729). Chromatin DNA was purified with protein G Dynabeads (Invitrogen, 10004D) and subjected to real-time PCR. The region-specific primers used are listed in Table S1.

## Results

### EPB41L4A-AS1 is downregulated in aging and neurodegenerative diseases

**Fig. 1:**
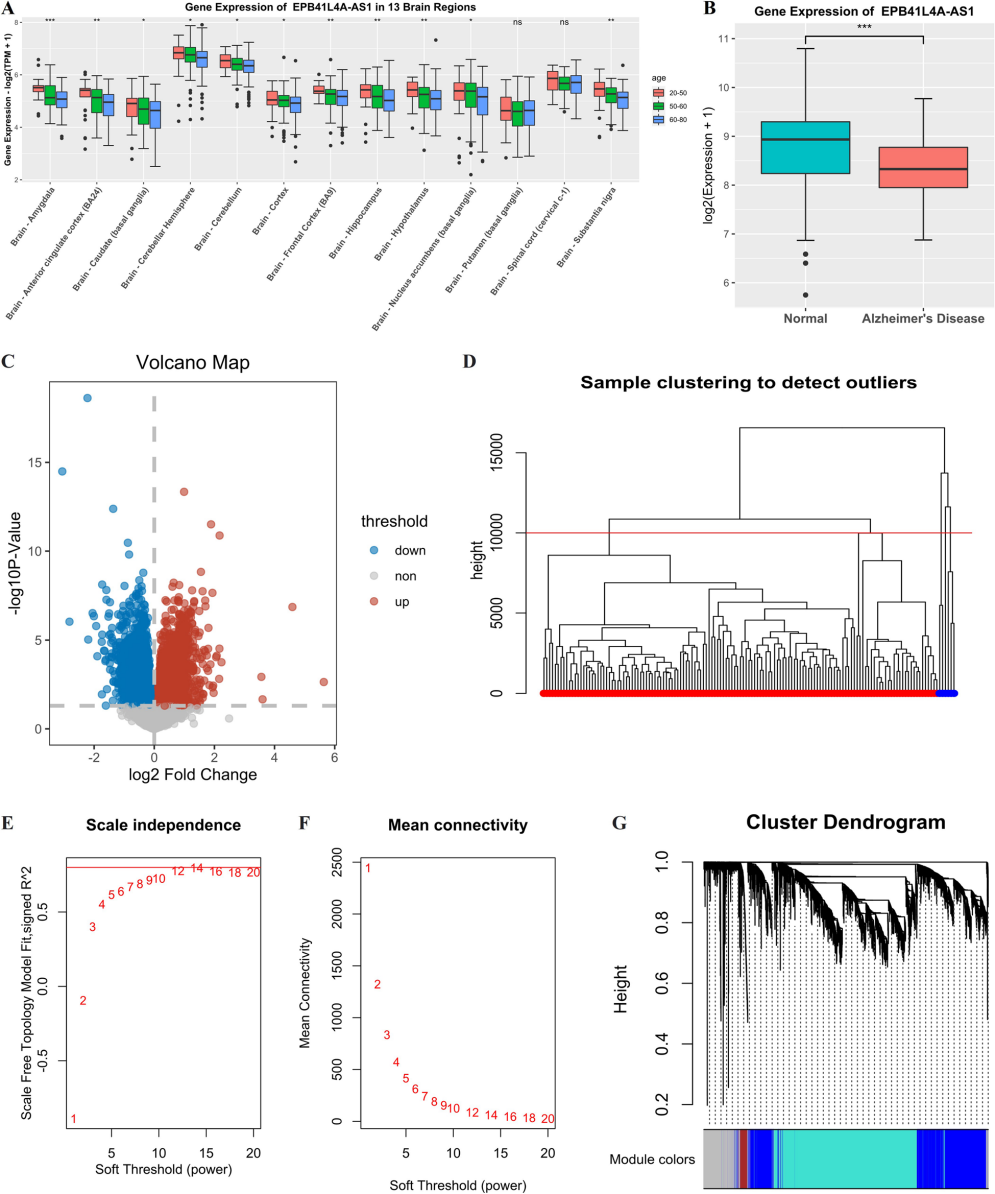
Gene expression of EPB41L4A-AS1, DEGs screening, and module clustering. **A** Gene expression of EPB41L4A-AS1 in 13 brain regions with different ages. Data are shown as median with interquartile range, *p < 0.05, **p < 0.01, ***p < 0.001, ****p < 0.0001. **B** Gene expression of EPB41L4A-AS1 in normal and Alzheimer's disease samples. **C** Volcano plot of genes in the hippocampus, blue, red, and grey dots represent downregulated, upregulated genes and genes without differential expression (the elderly group versus the young group). **D** Sample clustering to detect outliners, the height limit is 10000, and the samples in blue (height > 10,000) are outliers. **E** Scale independence of the scale-free networks, the limit of signed R^2^ of scale-free topology module fit is 0.8, and powers that make the R^2^ greater than 0.8 are appropriate for the networks. **F** Mean connectivity of the scale-free networks. **G** Cluster dendrogram of DEGs, the grey module holds the genes which were failed to cluster

Given that EPB41L4A-AS1 has been reported to be a metabolism-related lncRNA and altered brain metabolic patterns have been established in aging and neurodegenerative diseases, especially in AD, we hypothesized that the expression of EPB41L4A-AS1 could be dysregulated under these conditions. The Mann–Whitney U test compared EPB41L4A-AS1 expression in different age groups and between normal and AD groups. As shown in Fig. 1A, B, EPB41L4A-AS1 expression gradually decreased with aging and was downregulated in the AD group, compared with normal subjects.

### Differences of gene expression in the hippocampus of different age groups

It is widely acknowledged that the hippocampus is the main lesion site in aging and neurodegenerative diseases; accordingly, the gene expression profiles (32972 genes) of 197 samples from the hippocampus (20–50 years n = 31, 50–60 years n = 52, 60–80 years n = 114) were extracted for differential gene expression analysis (age-stratified). The expression profiles of two samples (20–50 years, 60–80 years) were used as an expression matrix to obtain better results.

A total of 6538 DEGs (3246 upregulated and 3292 down-regulated) were screened between the elderly (60–80 years) and the young (20–50 years) groups with a threshold p-value < 0.05. EPB41L4A-AS1 downregulation was also confirmed in a volcano plot of DEGs (Fig. 1C).

### Construction of co-expression modules

To identify genes with similar expression patterns to EPB41L4A-AS1, the expression profile of all DEGs in 197 hippocampal samples was extracted to construct a weighted co-expression network using the WGCNA algorithm. The appropriate samples were screened out using sample clustering (Fig. 1D), and the appropriate power value was screened out (Fig. 1E, F). Scale-free networks were constructed with proper independence degree and average connectivity when the power value was 14. DEGs with similar expression patterns were clustered into four distinct gene co-expression modules (Fig. 1G).

### Interaction analysis with clinical traits and GO, KEGG enrichment

**Fig. 2:**
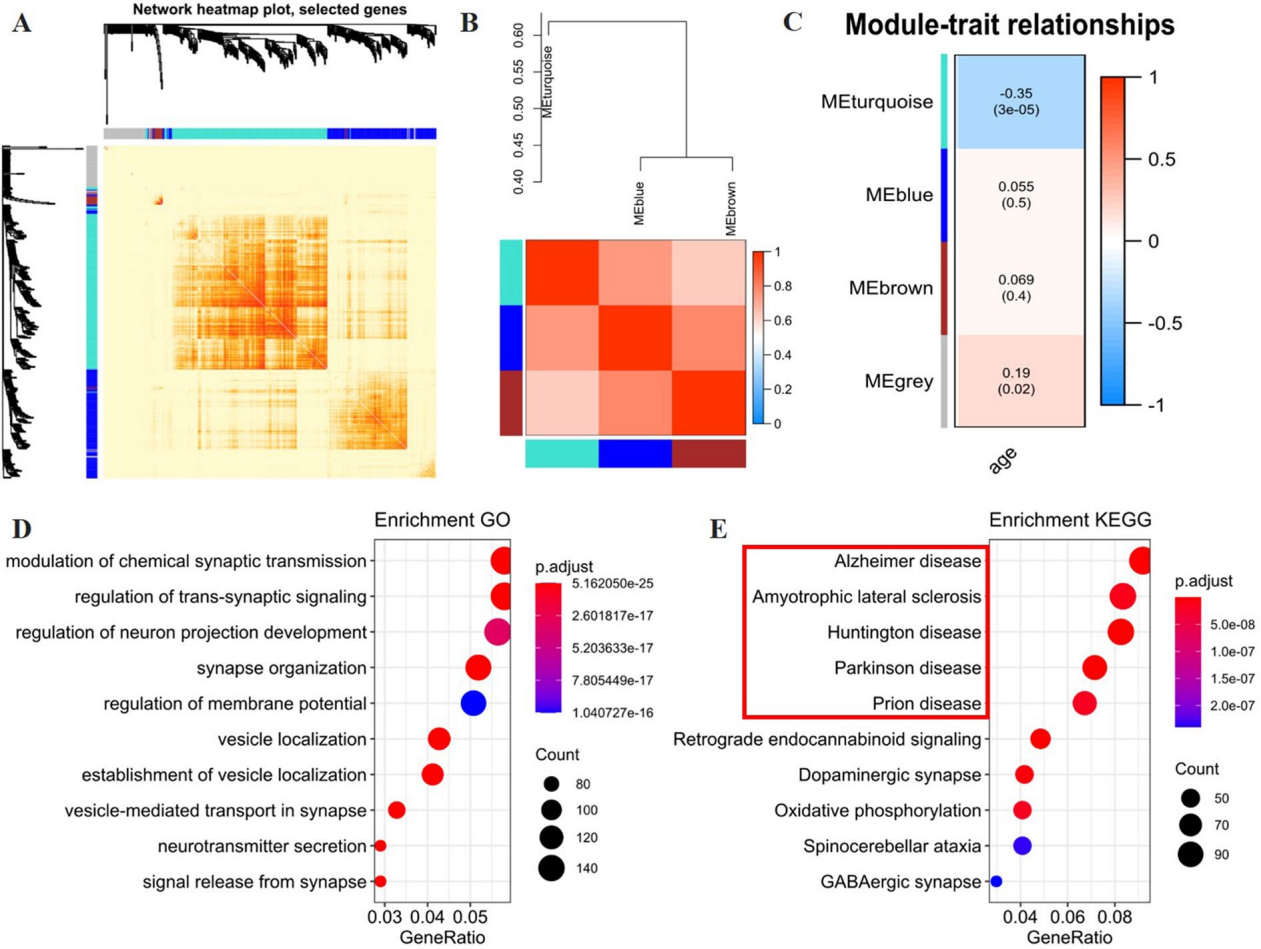
Function analysis of EPB41L4A-AS1 related genes. **a** TOM plot of 400 selected genes. **b** Correlation of different modules. **c** Correlation of modules and age. **d** Enrichment GO of the turquoise module. **e** Enrichment KEGG of the turquoise module

In our analysis, a heatmap (Fig. 2A) was generated to demonstrate the Topological Overlap Matrix (TOM) among 400 randomly selected genes. The correlation of three modules successfully clustered is shown in the heatmap (Fig. 2B). As a result, each module is independent of the other.

EPB4L4A-AS1 was located in the turquoise module; accordingly, we hypothesized that EPB41L4A-AS1 could regulate the protein-coding genes associated with the turquoise module. At the same time, module-trait relationships (Fig. 2C) showed a negative correlation between the genes in the turquoise module and age (r = − 0.35, p < 0.001).

To obtain the functions of the turquoise module, GO and KEGG enrichment analyses were applied to all genes in the turquoise module. The top 10 GO terms and KEGG pathways are shown in Fig. 2D, E. Genes in the turquoise module were mainly enriched in nerve excitement transmission and neurodegenerative diseases (such as Alzheimer's disease (AD), amyotrophic lateral sclerosis (ALS), Huntington's disease (HD), Parkinson's disease (PD), and Prion disease) pathway.

### Correlation of EPB41L4A-AS1 and Protein-coding Genes (PCGs)

**Fig. 3:**
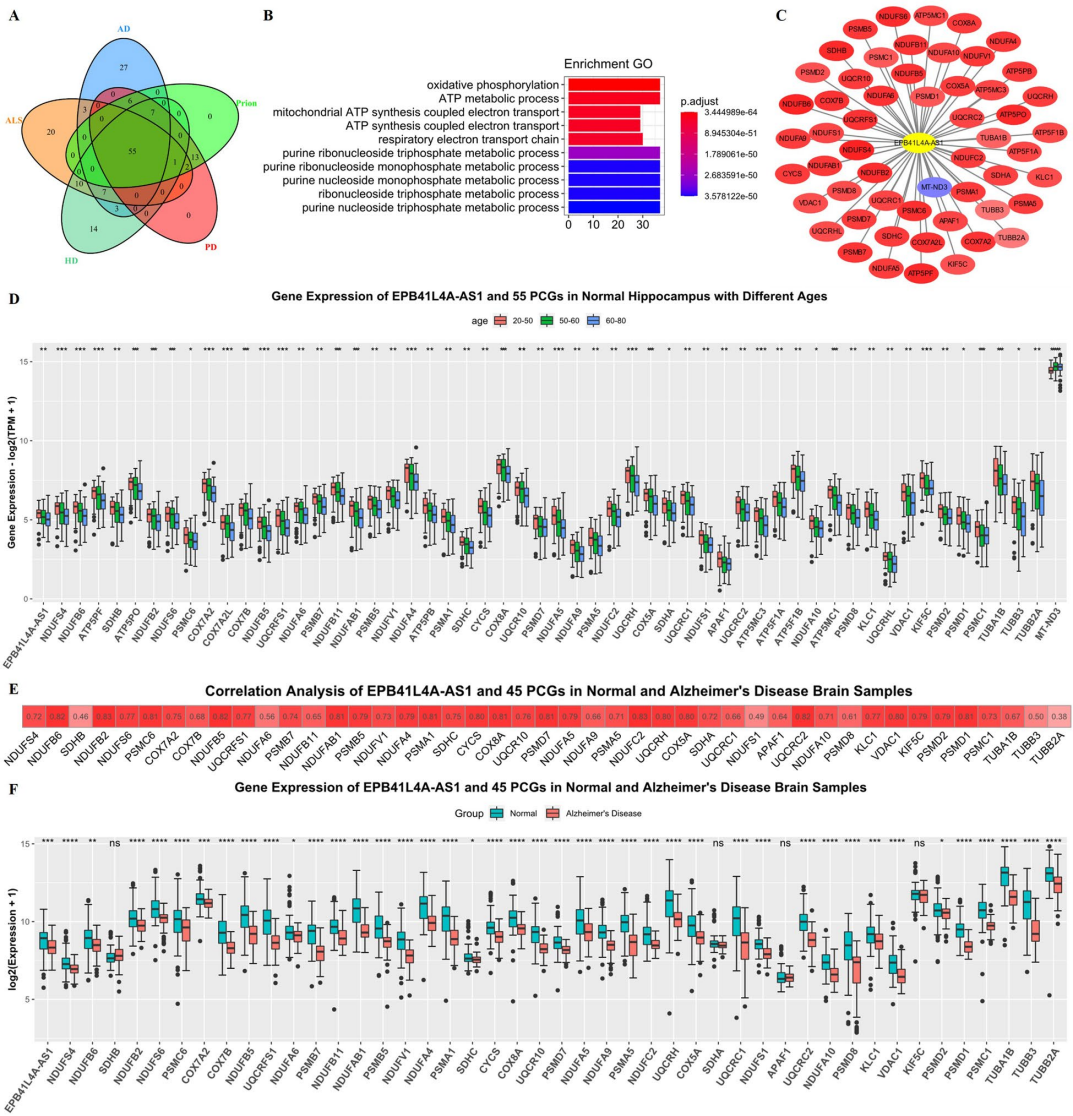
Screening of 55 PCGs and correlation between EPB41L4A-AS1 and 55 PCGs in the hippocampus. **A** The intersection of 5 neurodegenerative disease pathway genes. **B** Enrichment GO of 55 PCGs. **C** Correlation of EPB41L4A-AS1 and 55 PCGs in the hippocampus, the values of the correlation coefficients are also represented by color, red color represents a positive correlation, and blue color represents a negative correlation. **D** Gene expression of EPB41L4A-AS1 and 55 PCGs in the normal hippocampus with different ages, data were expressed as median with interquartile range, *p < 0.05, **p < 0.01, ***p < 0.001, ****p < 0.0001. **E** Correlation between EPB41L4A-AS1 and 45 PCGs in normal and AD samples. **F** Gene expression of EPB41L4A-AS1 and 45 PCGs in normal and AD samples

The DEGs of five neurodegenerative disease-related pathways were intersected, yielding a total of 55 PCGs (Fig. 3A). Further GO enrichment analysis of these 55 genes showed significant enrichment in "oxidative phosphorylation", "ATP metabolic process", and "electron transport" (Fig. 3B). We hypothesized that these 55 PCGs played important roles in aging and neurodegenerative diseases. Cluster analysis showed similar expression patterns between EPB41L4A-AS1 and these PCGs. Correlation analysis showed that EPB41L4A-AS1 was positively correlated with EPB41L4A-AS1 and the 55 hippocampal PCGS (Fig. 3C); these 55 PCGs were downregulated with brain aging (Fig. 3D). To prove the universality of this relationship, the correlation analysis results of the 13 brain regions were displayed in a heatmap (Additional file 1: Fig. S1A). Furthermore, analysis of EPB41L4A-AS1 expression and 55 PCGs fold changes in 13 brain regions showed that EPB41L4A-AS1 and 55 PCGs were universally downregulated in the brain with aging (Additional file 1: Fig. S1.B), substantiating that this relationship was universal.

### Gene function verification in AD dataset

GSE5281 dataset was used to investigate similarities in the expression pattern of EPB41L4A-AS1 related genes between the aging group and the AD neurodegenerative diseases groups. For technical reasons, the expression data of only 45 out of 55 PCGs were analyzed. Correlation analysis showed a strong positive correlation between EPB41L4A-AS1 and the 45 PCGs(Fig. 3E). Moreover, EPB41L4A-AS1 and these PCGs were downregulated in AD samples (Fig. 3F). These results further substantiated our hypothesis.

### Protein–protein interaction network of 55 PCGs

**Fig. 4:**
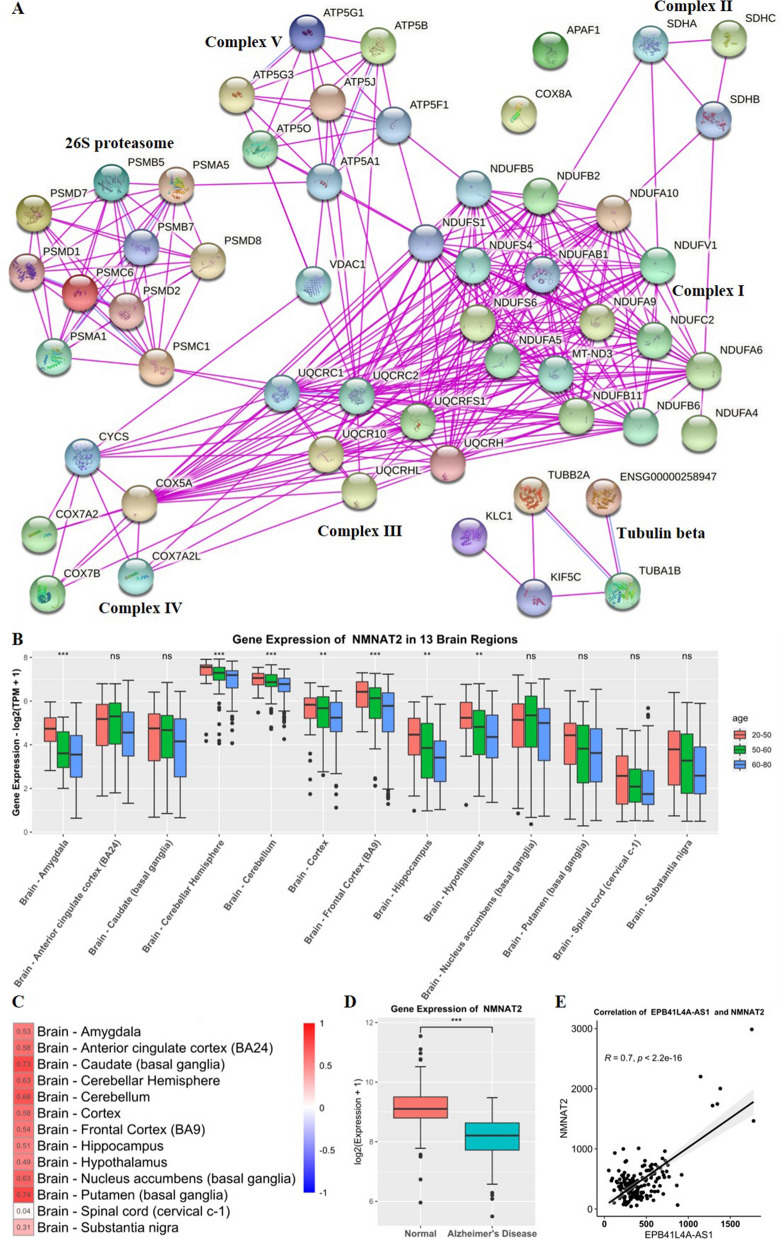
PPI network for 55 PCGs and analysis of the regulation of EPB41L4A-AS1 on NMNAT2. **A** PPI network for 55 PCGs from experiments, 55 PCGs are main components of complex I—complex V. **B** Gene expression of NMNAT2 in 13 brain regions with different ages. **C** Correlation of EPB41L4A-AS1 and NMNAT2 in 13 brain regions, the values of the correlation coefficients are also represented by color; red color represents a positive correlation while blue color represents a negative correlation. **D** Gene expression of NMNAT2 in normal and AD samples. **E** Correlation of EPB41L4A-AS1 and NMNAT2 in normal and AD samples

The 55 PCGs were uploaded to STRING to generate a protein–protein interaction network (Fig. 4A). Molecular biological analysis showed that the 55 PCGs were the main components of respiratory electron transport chain complexes I-V, which participate in the third stage of cellular respiration. Complex I is known to catalyze the production of NAD^+^ from NADH [24, 25], while complex I-V can catalyze the synthesis of ATP from ADP by affecting the electron transport chain [26] [27].

At this stage, we found a strong positive correlation between EPB41L4A-AS1 and 55 PCGs, and these 55 genes were found to be involved in the synthesis of respiratory electron transport chain complexes I-V. By analyzing the reactions associated with the electron transport chain, we hypothesized that EPB41L4A-AS1 downregulation could downregulate the expression of complexes I-V components, thus reducing the activity of complexes I-V and ultimately leading to the downregulation of NAD^+^ and ATP levels.

Previous studies have reported that brain NAD^+^ was associated with ATP synthesis and membrane phospholipid turnover in humans [28], while ATP played a very important role in the normal brain [29]. Furthermore, NAD^+^ has been strongly associated with aging and neurodegenerative diseases [18, 30], with evidence suggesting NAD^+^ can inhibit Alzheimer's disease [31]. As a result, a decrease in NAD^+^ and ATP levels is often paralleled by a decrease in the body's energy levels and anti-aging ability, eventually leading to aging and neurodegenerative diseases [32].

### Analysis of NAD^+^ synthesis pathway genes

It is widely acknowledged that the electron transport chain is a major contributor to NAD^+^ levels, which are also sustained by three independent biosynthetic pathways, kynurenine (or de novo synthesis pathway), Preiss–Handler, and salvage pathways [16]. Interestingly, the kynurenine pathway has been associated with neurodegenerative diseases [33, 34], while the salvage pathway was found to protect neurons from chemotherapy-induced degeneration [35]. Inspired by these findings, we analyzed the expression levels of genes in the NAD^+^ biosynthesis pathways in aging and neurodegenerative diseases and found that NMNAT2, previously documented to be enriched in the cytoplasm [36], was mainly expressed in the brain (Additional file 2: Fig. S2A). Importantly, it has been shown that NMNATs could catalyze the reaction of NAMN (Preiss–Handler pathway) and NMN (salvage pathway) to form NAD^+^, and NMNAT2 is the predominant form of NMNATs in the brain (Additional file 2: Fig. S2B). In addition, NMNAT2 was found in the same module (turquoise module) as EPB41L4A-AS1 during WGCNA and downregulated with aging (Fig. 4B). Correlation analysis showed that EPB41L4A-AS1 exerted a strong positive regulation on NMNAT2 in aging (Fig. 4C). We further analyzed NMNAT2 expression in normal and AD samples and its correlation with EPB41L4A-AS1 in dataset GSE5281. As shown in Fig. 4D, E, NMNAT2 was downregulated in Alzheimer's disease and strongly correlated with EPB41L4A-AS1.

In short, EPB41L4A-AS1 could regulate NAD^+^ and ATP synthesis by impacting the expression of genes associated with the electron transport chain and NAD^+^ synthesis pathway (NMNAT2), thus playing an important role in brain aging and neurodegenerative diseases. The detailed mechanism is shown in Additional file 2: Fig. S2C.

### EPB41L4A-AS1 has a positive regulation of NAD^+^ and ATP synthesis in SH-SYSY and U251 cells

In the present study, the role and mechanism of EPB41L4A-AS1 in brain aging and neurodegenerative diseases were assessed in SH-SY5Y and U251 cells. For each mitochondrial respiratory chain complex, two PCGs with the highest correlation coefficient with EPB41L4A-AS1 were selected for further analysis. As a result, a total of 11 PCGs (10 genes of 5 complexes, NMNAT2) were selected to validate if EPB41L4A-AS1 could regulate their expression.

**Fig. 5:**
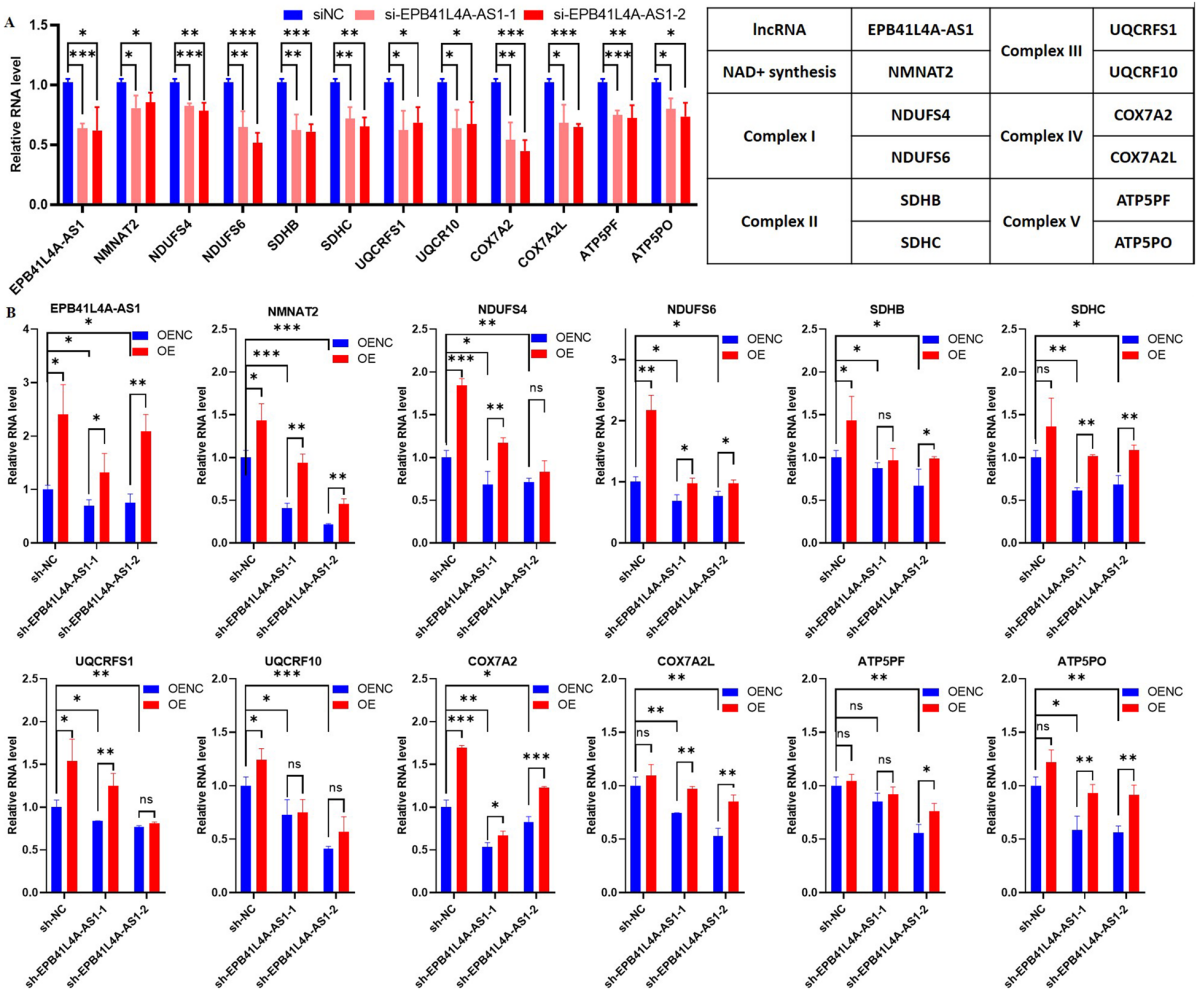
EPB41L4A-AS1 regulates the expression of 11 PCGs in SH-SY5Y cells. **A** Relative RNA expression of genes in si-EPB41L4A-AS1 and siNC cells. **B** Relative RNA expression of genes in sh-EPB41L4A-AS1 and sh-NC cells, *OENC* cells transfected with empty plasmids, *OE* cells transfected with EPB41L4A-AS1 overexpression plasmids. Data are shown as mean ± SD. *p < 0.05, **p < 0.01, ***p < 0.001, ****p < 0.0001, student’s t-test

First, siRNAs were used to knock down EPB41L4A-AS1 in SH-SY5Y cells and observe the changes in the 11 PCGs. We found that two siRNAs (si-EPB41L4A-AS1-1, si-EPB41L4A-AS1-2) were successfully transiently transfected, and the relative mRNA expressions of the 11 target genes were downregulated (Fig. 5A). The lentiviral vector EPB41L4A-AS1 was transfected to obtain stable EPB41L4A-AS1 knockdown cell lines to assess the stability of changes in the 11 target genes when EPB41L4A-AS1 was knocked down. Two sh-EPB41L4A-AS1 cell lines (sh-EPB41L4A-AS1-1, sh-EPB41L4A-AS1-2) were obtained, yielding similar results to the transient transfection findings (Fig. 5B). Finally, the EPB41L4A-AS1 overexpression plasmids were successfully transfected in the sh-NC cell line and two sh-EPB41L4A-AS1 cell lines. As shown in Fig. 5B, a "rebound" increase in expression levels of the 11 PCGs was observed when EPB41L4A-AS1 was overexpressed.

To increase the reliability of our cell experiments, the above experiments were repeated in U251 cells. As shown in Additional file 3: Fig. S3, the expressions of 11 target genes were downregulated when siRNAs were transiently transfected (Additional file 3: Fig. S3A), and sh-EPB41L4A-AS1 lentivirus transfection (Additional file 3Fig. S3B). Consistently, the expression of the 11 target genes was upregulated as EPB41L4A-AS1 was overexpressed (Additional file 3: Fig. S3B).

**Fig. 6:**
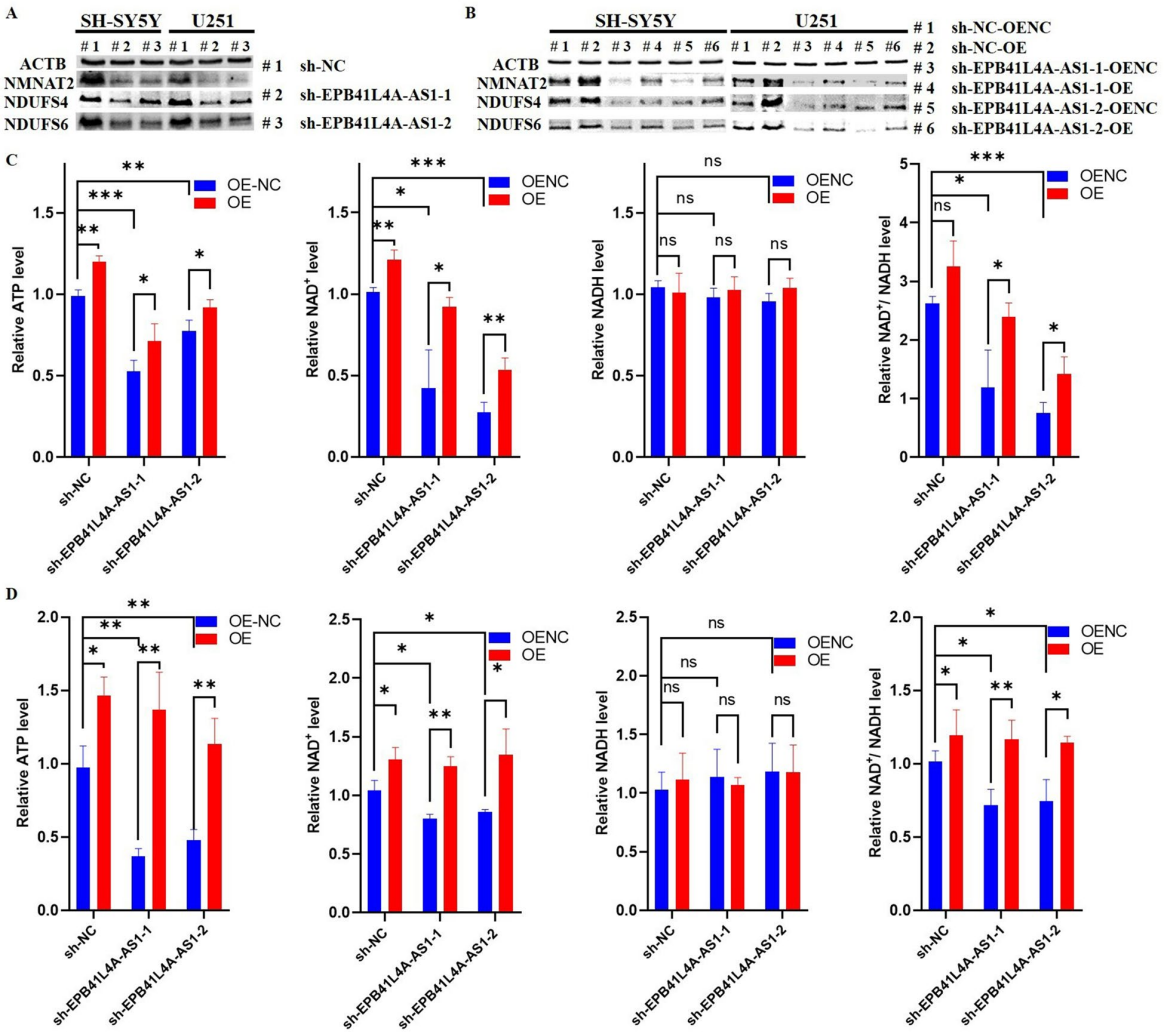
Western blot and measurement of ATP, NAD^+^, and NADH levels. (A-B) Western blot analysis for NAD^+^ and ATP synthesis genes when EPB41L4A-AS1 is knocked down (**A**) and overexpressed (**B**) in SH-SY5Y and U251 cells, ACTB as the internal reference gene. **C**, **D** Relative ATP, NAD^+^, NADH, and NAD^+^/NADH levels in EPB41L4A-AS1 knocked down or overexpressed SY5Y (**C**) and U251 (**D**) cells, *OENC* cells transfected with empty plasmids, *OE* cells transfected with EPB41L4A-AS1 overexpression plasmids

Subsequently, Western blot analysis was used to quantify the protein levels of the NAD^+^ and ATP synthesis-related genes in SH-SY5Y and U251 cells. For ATP synthesis-related genes, NDUFS4 and NDUFS6 were the representative genes since they constituted a significant proportion of genes in complex I during the initial bioinformatics analysis. Accordingly, the protein levels of NMNAT2, NDUFS4, and NDUFS6 were quantified. As shown in Fig. 6A, B, the protein levels of these genes were downregulated by EPB41L4A-AS1 knockdown and upregulated with EPB41L4A-AS1 overexpression.

We finally measured NAD^+^, NADH, and ATP levels in SH-SY5Y and U251 cells. As shown in Fig. 6C and D, no significant change in NADH was observed in sh-EPB41L4A-AS1 cells while the NAD^+^, NAD^+^/NADH ratio, and ATP levels were downregulated and upregulated by overexpression of EPB41L4A-AS1. So far, we have validated our hypothesis on the regulatory role of EPB41L4A-AS1 at the bioinformatics, molecular biology, and cell biology levels.

### NR treatment can improve the NAD^+^ and ATP levels in SH-SY5Y and U251 cells

**Fig. 7:**
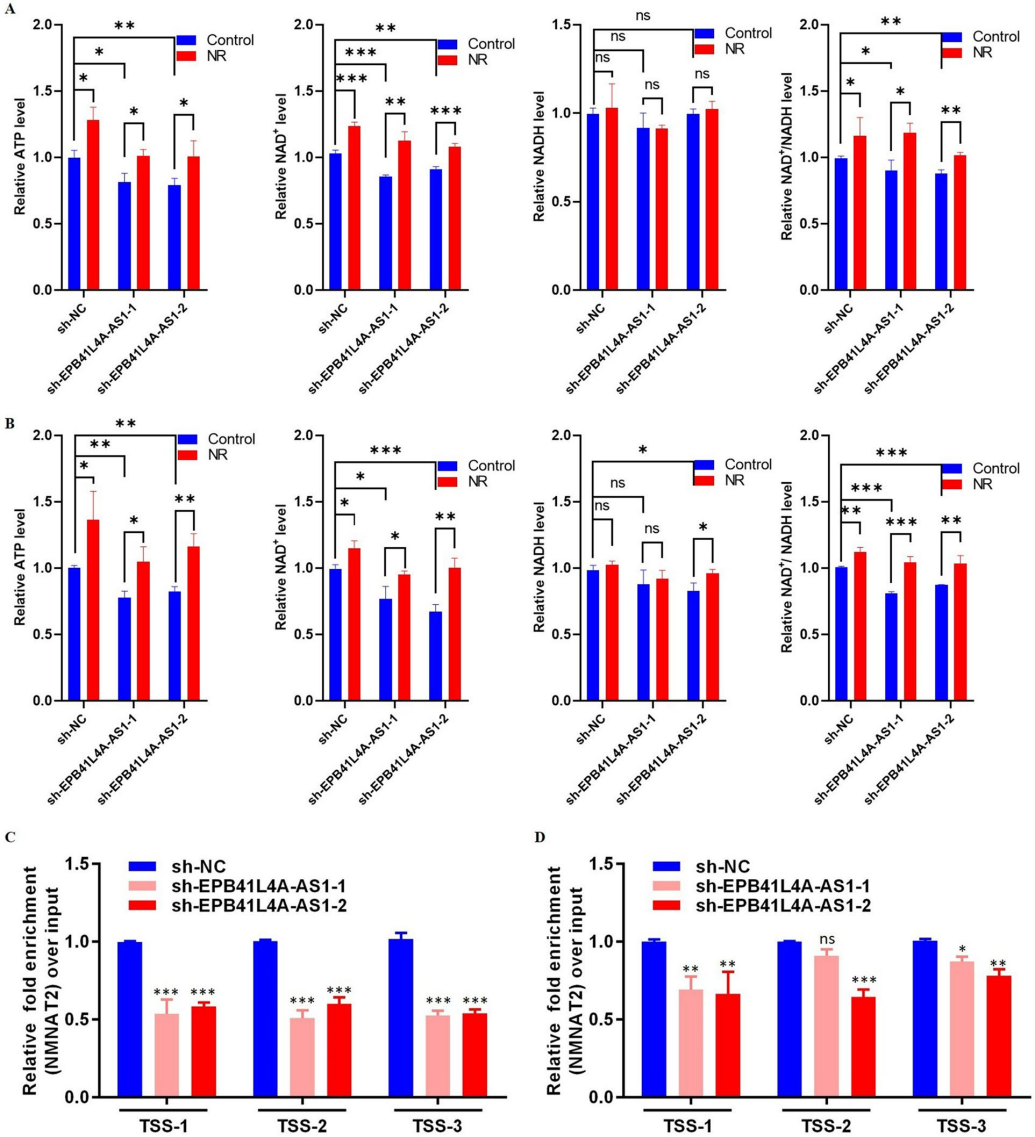
NR treatment and quantification of acetylated histones. **A**, **B** Relative ATP, NAD^+^, NADH, and NAD^+^/NADH levels in NR treatment SY5Y (**A**) and U251 (**B**) cells, NR: cells treatment with NR, Control: cells without NR treatment. **C**, **D** H3K27Ac levels to the transcription start site (TSS) of NMNAT2 in SH-SY5Y (**C**) and U251 (**D**) cells. Data are shown as mean ± SD. *p < 0.05, **p < 0.01, ***p < 0.001, ****p < 0.0001, student’s t-test

Given that overexpression of EPB41L4A-AS1 has been proved to restore ATP and NAD^+^ levels and NAD^+^ depletion has been established to be a contributing factor for mitochondrial dysfunction, which reduces ATP synthesis, rescue experiments were performed; SH-SY5Y and U251 cells were treated with NR (nicotinamide riboside, a substrate of NAD^+^ synthesis) to assess whether NR could alleviate decreased NAD^+^ synthesis induced by EPB41L4A-AS1 downregulation, and further alleviate the decrease in ATP synthesis caused by decreased NAD^+^ synthesis. As shown in Fig. 7A and B, a"rebound" increase in NAD^+^ and ATP levels was observed after NR treatment in SH-SY5Y and U251 cells. Importantly, our results showed that NR and EPB41L4A-AS1 could both increase ATP and NAD^+^ levels.

### EPB41L4A-AS1 has a positive regulation of the acetylation of histones

It has been reported that EPB41L4A-AS1, functioning as a lncRNA, could regulate gene expression at the epigenetic level by mainly affecting the acetylation of histones [7]. A chromatin immunoprecipitation (ChIP) assay was conducted in the present study to quantify H3K27Ac levels at the NMNAT2 gene promoter in SH-SY5Y and U251 cells. As shown in Fig. 7C, D, EPB41L4A-AS1 inhibition downregulated H3K27Ac near the transcription start site (TSS) of NMNAT2. This result accounted for the regulatory mechanism of EPB41L4A-AS1 at the epigenetic level.

## Discussion

In the present study, we first performed a bioinformatics analysis to reveal the mechanisms of lncRNA EPB41L4A-AS1 in brain aging. Analysis of GTEx and GEO datasets showed that EPB41L4A-AS1 expression was downregulated in the aging brain and AD group, compared with normal people. Then, we compared the hippocampal gene expression between the elderly and the young group and constructed co-expression modules. DEGs with similar expression patterns were clustered into four distinct gene co-expression modules. EPB4L4A-AS1 was located in the turquoise module, and genes in the turquoise module were associated with aging and neurodegenerative diseases. Previous studies have shown that the occurrence of neurodegenerative diseases was closely related to changes in oxidative phosphorylation [37, 38], which further illustrated the importance of identified genes in the turquoise module.

In the turquoise module, 55 genes were obtained after intersecting five datasets of different neurodegenerative diseases. Correlation analysis of EPB41L4A-AS1 and 55 PCGs in 13 brain regions found that EPB41L4A-AS1 exerted a strong positive regulatory effect on these genes. Subsequently, we validated the regulatory role of EPB41L4A-AS1 using the AD dataset GSE5281, which yielded consistent results. Furthermore, functional analysis of these 55 genes showed that these genes were highly related to the electron transport chain and ATP synthesis in the cell respiration process. As seen in the PPI network, these genes were mainly involved in forming mitochondrial respiratory complexes I-V. All five complexes could affect ATP synthesis, and among them, complex I comprised the largest number of genes, while complex I was mainly involved in the conversion of NADH to NAD^+^. Given that NAD^+^ is widely acknowledged to play in mitochondrial respiration, we analyzed the NAD^+^ synthesis pathway genes and found that NMNAT2 was down-regulated DEG in aging and Alzheimer's disease. Moreover, NMNAT2 was highly correlated with EPB41L4A-AS1 both in aging and Alzheimer's disease. Accordingly, we substantiated that EPB41L4A-AS1 downregulation mediated brain aging and the development of neurodegenerative diseases via decreasing NAD^+^ and ATP synthesis.

Finally, we validated the regulatory mechanisms by cell experiments on U251 and SH-SY5Y cells. As predicted by our in silico analysis, genes regulated by EPB41L4A-AS1 were downregulated when EPB41L4A-AS1 was knocked down, with decreased NAD^+^ and ATP levels. Importantly, opposite findings were observed once EPB41L4A-AS1 was overexpressed. Furthermore, the NR treatment experiment shows the positive significance of EPB41L4A-AS1 in treatment, while experiments at the epigenetic level showed that the regulatory mechanism of EPB41L4A-AS1 was based on mediating acetylated histone H3 Lys24 (H3K24ac) levels.

NAD^+^ is a critical metabolite and coenzyme that is always in high demand. Sustaining its stable intracellular level is important for the normal function of multiple metabolic pathways and cellular processes. NAD^+^ level is maintained by three independent biosynthetic pathways, including the kynurenine, the Preiss–Handler, and the NAM salvage pathways [16, 39, 40]. The balance of NAD^+^ between synthesis and degradation can be disrupted during aging; however, the underlying mechanism remains unclear [16]. This study found that NMNAT2, a brain-specific nicotinamide mononucleotide adenylyltransferase, was downregulated by EPB41L4A-AS1. NMNATs were involved in the Preiss–Handler and the NAM salvage pathways, and its decrease with age could be the cause of the decrease in NAD^+^ levels in the aging brain. It has been reported that NMNAT2 depletion was related to SARM1-dependent axon degeneration in Parkinson's disease and other axonal disorders [41].

An increasing body of evidence suggests that brain NAD^+^ levels play a central role in maintaining energy homeostasis and ATP production [28]. In this study, we found that the aging-induced down-regulation of EPB41L4A-AS1 not only disturbed NAD^+^ biosynthesis but also affected ATP production. It has been established that NAD^+^ accepts hydride equivalents, forming NADH, providing electrons to the electron transport chain to generate ATP [28]. In this respect, a decrease of NAD^+^ could disrupt the process and impact ATP synthesis. Importantly, EPB41L4A-AS1 downregulation could inhibit the expression of genes related to the electron transport chain, causing a decrease in ATP synthesis. Under such conditions, the high demand of the brain for NAD^+^ and ATP cannot be met, promoting the development of brain aging and neurodegenerative diseases.

## Conclusion

In conclusion, our study reported that EPB41L4A-AS1 exerted a strong positive regulatory effect on genes that form the respiratory electron transport chain complexes I-V and genes in NAD^+^ biosynthetic pathways. EPB41L4A-AS1 downregulation mediated by aging could lead to decreased expression of these genes and decreased NAD^+^ and ATP levels. Accordingly, many normal physiological functions could be inhibited, impacting normal metabolism and resulting in aging and the onset of neurodegenerative diseases. Importantly, these symptoms were relieved when EPB41L4A-AS1 was overexpressed, providing a new approach for anti-aging and treating neurodegenerative diseases.

## Supplementary Information

**Additional file 1: Fig. S1**. (A) Correlation analysis of EPB41L4A-AS1 and 55 PCGs in 13 brains regions. (B) Fold changes in gene expression of EPB41L4A-AS1 and 55 PCGs in 13 brain regions, the fold changes are calculated by Mean (Old group, 60-80 years) / Mean(Young group, 20-50 years).

**Additional file 2: Fig. S2**. (A) Gene expression for NMNAT2 in 54 normal tissues. (B) Gene expression of NMNATs in 13 brain regions. (C) The mechanism of lncRNA EPB41L4A-AS1 in regulating brain metabolism, lncRNA EPB41L4A-AS1 regulates NAD+ and ATP levels by affecting the complex I-V genes NAD+ synthesis pathway gene NMNAT2.

**Additional file 3: Fig. S3**. EPB41L4A-AS1 regulates the expression of 11 PCGs in U251 cells. (A) Relative RNA expression of genes in si-EPB41L4A-AS1 and siNC cells. (B) Relative RNA expression of genes in sh-EPB41L4A-AS1 and shNC cells, OENC: cells transfected with empty plasmids, OE: cells transfected with EPB41L4A-AS1 overexpression plasmids. Data are shown as mean ± SD. *p<0.05, **p<0.01, ***p<0.001, ****p<0.0001, student’s t-test.

**Additional file 4: Table S1**. List of the primer sequences for real-time quantitative PCR.

## Data Availability

All data analyzed during this study are included in this article.

## Abbreviations

lncRNA: Long noncoding RNA
GTEx: Genotype-Tissue Expression
DEGs: Differentially expressed genes
WGCNA: Weighted correlation network analysis
GO: Gene Ontology
KEGG: Kyoto Encyclopedia of Genes and Genomes
PPI: Protein–protein interaction
GEO: Gene Expression Omnibus
NR: Nicotinamide riboside
Chip: Chromatin Immunoprecipitation
H3K27Ac: Acetylation of lysine 27 on histone 3
PCG: Protein-coding gene
AD: Alzheimer’s disease
ALS: Amyotrophic lateral sclerosis
HD: Huntington’s disease
PD: Parkinson’s disease
TSS: Transcription start site
