# Supplementary figures and images for: Down-regulation of EPB41L4A-AS1 mediated the brain aging and neurodegenerative diseases via damaging synthesis of NAD+ and ATP
Source: Cell Biosci. 2021 Nov 10;11:192. doi: 10.1186/s13578-021-00705-2 (PMC8579638; doi:10.1186/s13578-021-00705-2)

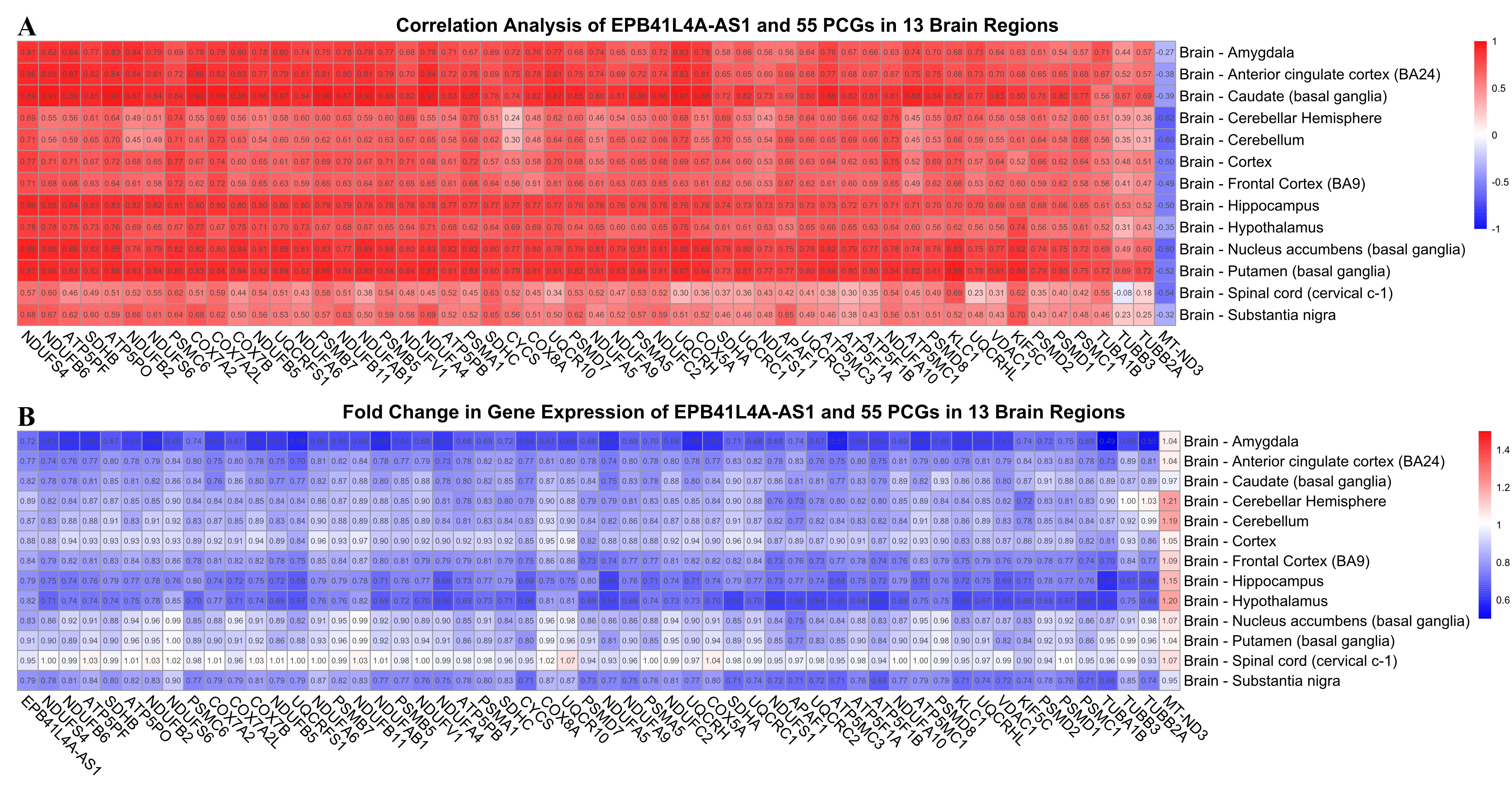

Supplement: Supplementary file 1 — Additional file 1: Fig. S1. (A) Correlation analysis of EPB41L4A-AS1 and 55 PCGs in 13 brains regions. (B) Fold changes in gene expression of EPB41L4A-AS1 and 55 PCGs in 13 brain regions, the fold changes are calculated by Mean (Old group, 60-80 years) / Mean(Young group, 20-50 years). [file 13578_2021_705_MOESM1_ESM.jpg]

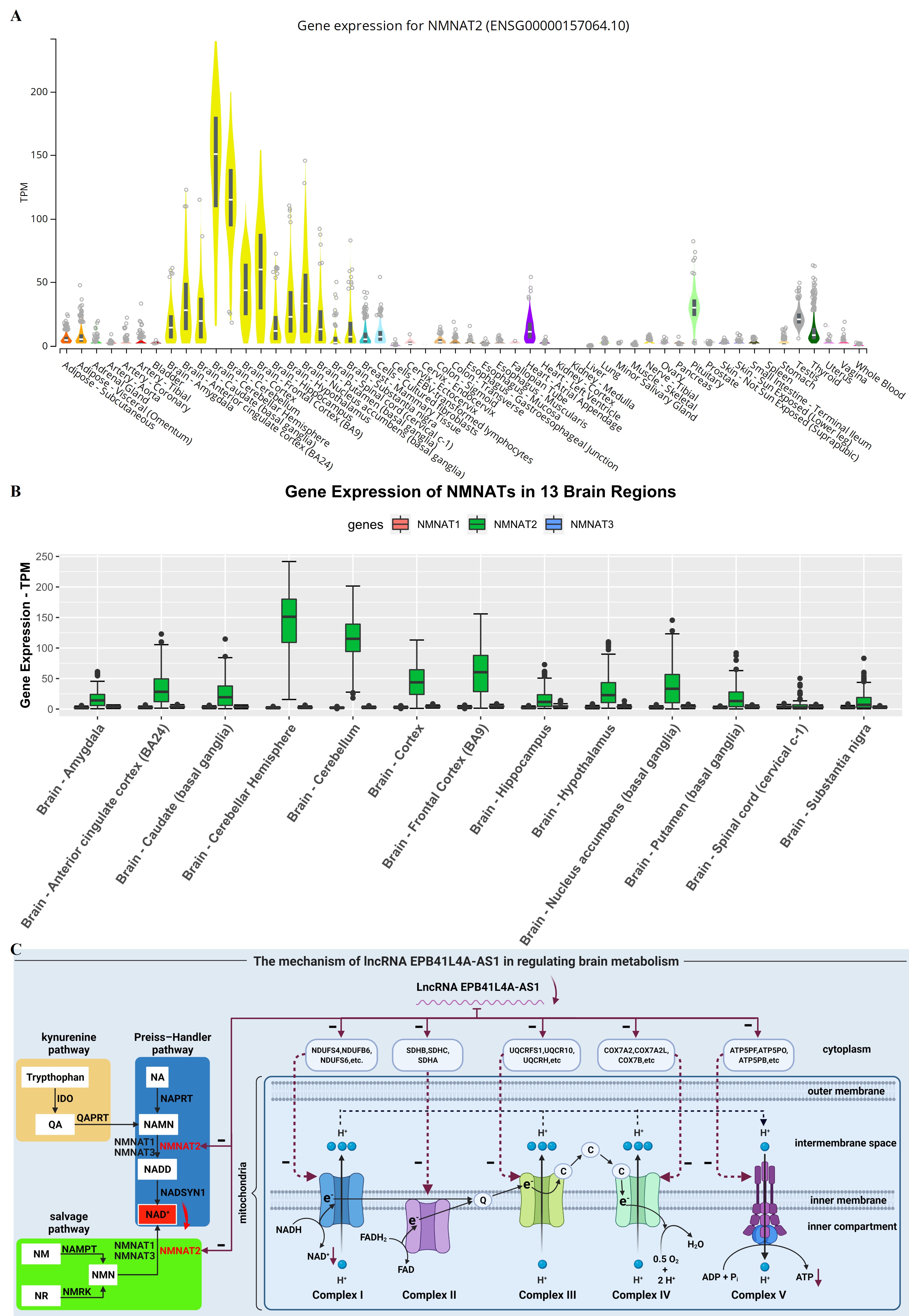

Supplement: Supplementary file 2 — Additional file 2: Fig. S2. (A) Gene expression for NMNAT2 in 54 normal tissues. (B) Gene expression of NMNATs in 13 brain regions. (C) The mechanism of lncRNA EPB41L4A-AS1 in regulating brain metabolism, lncRNA EPB41L4A-AS1 regulates NAD+ and ATP levels by affecting the complex I-V genes NAD+ synthesis pathway gene NMNAT2. [file 13578_2021_705_MOESM2_ESM.jpg]

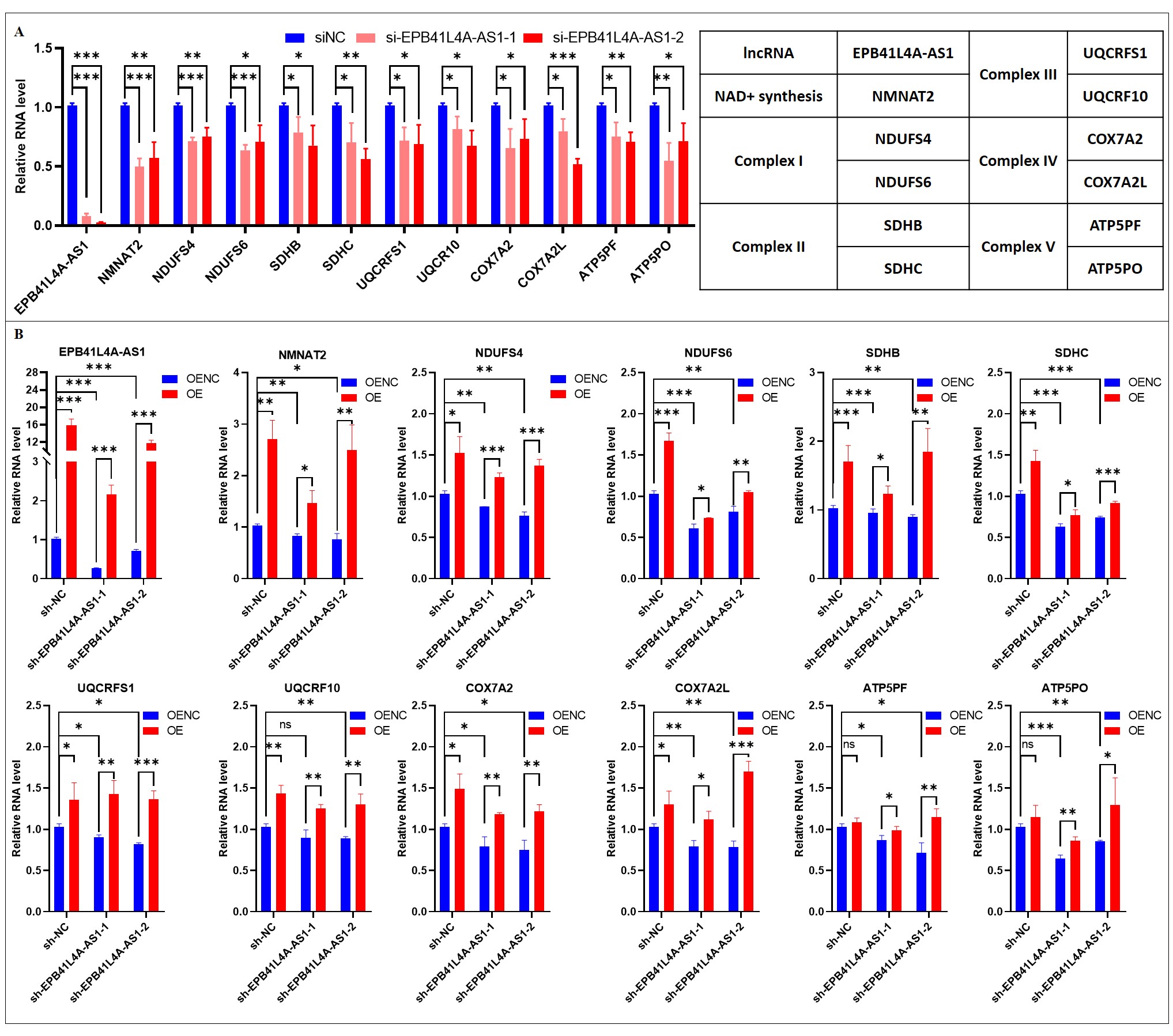

Supplement: Supplementary file 3 — Additional file 3: Fig. S3. EPB41L4A-AS1 regulates the expression of 11 PCGs in U251 cells. (A) Relative RNA expression of genes in si-EPB41L4A-AS1 and siNC cells. (B) Relative RNA expression of genes in sh-EPB41L4A-AS1 and shNC cells, OENC: cells transfected with empty plasmids, OE: cells transfected with EPB41L4A-AS1 overexpression plasmids. Data are shown as mean ± SD. *p<0.05, **p<0.01, ***p<0.001, ****p<0.0001, student’s t-test. [file 13578_2021_705_MOESM3_ESM.jpg]
