# Supplementary material for: Down-regulation of EPB41L4A-AS1 mediated the brain aging and neurodegenerative diseases via damaging synthesis of NAD+ and ATP
Source: Cell Biosci. 2021 Nov 10;11:192. doi: 10.1186/s13578-021-00705-2 (PMC8579638; doi:10.1186/s13578-021-00705-2)
Supplement: Supplementary file 4 — Additional file 4: Table S1. List of the primer sequences for real-time quantitative PCR. [file 13578_2021_705_MOESM4_ESM.docx]

|  | Forward | Reverse |
| --- | --- | --- |
| ACTB | TGACGTGGACATCCGCAAAG | CTGGAAGGTGGACAGCGAGG |
| U6 | CTCGCTTCGGCAGCACA | AACGCTTCACGAATTTGCGT |
| EPB41L4A-AS1 | CCTGGTTTTATTTTCGTCA | ATCCATCTTCCACCTGTAG |
| NDUFS4 | AGACGTTGTGGCGGAGAAG | GAGTCTGGTCCTGTGCCAAT |
| NDUFS6 | CTGTGGGCTCCAGTTCAGAC | GCGGAAATGCTCACAGGATG |
| SDHB | TTGCACCCGAAGGATTGACA | GCTGCTTGCCTTCCTGAGAT |
| SDHC | GTGGGGGTGGAGGGGAATTA | ATCTTCCTCGTGTCCAAAGCTA |
| UQCRFS1 | ACCCAGTTCGTTTCCAGCAT | CAGGGGTTTGCCTCTCCATT |
| UQCR10 | GAATTGCCCTTGAGACCTGCT | TTCTGCCTTTGGGGTGTTTG |
| COX7A2 | CCAGCAATCGCTTGGTTCAG | GACTGCTTTATTGGTGGCAGT |
| COX7A2L | TTCCACAGAAGCACCACCTA | TAGCTCTGGAACTTTGTTTTTCCC |
| ATP5PF | ATCTGGAGGACCTGTTGATGC | TGTATTCATGTCTGCATTACCAAAC |
| ATP5PO | GAACCCAAAGTGGCTGCTTC | TGGTAGTGAGGGGAGAGAACC |
| NMNAT2-TSS-1 | TGGTGTGAGCTCTTTCTGCC | GATAGCATTGCTTCGCCCAC |
| NMNAT2-TSS-2 | GTTCGATCGCCCTGGAAAAC | AGAGAGGCAACCCCTAGACC |
| NMNAT2-TSS-3 | TTCACTCAGCATCCACCTCG | GGCTGGAGATAACGTGCTGT |
